# A Review of Distributed Optical Fiber Sensors for Civil Engineering Applications

**DOI:** 10.3390/s16050748

**Published:** 2016-05-23

**Authors:** António Barrias, Joan R. Casas, Sergi Villalba

**Affiliations:** 1 Department of Civil and Environmental Engineering, Technical University of Catalonia (UPC), c/ Jordi Girona 1-3, Barcelona 08034, Spain; joan.ramon.casas@upc.edu; 2 Department of Engineering and Construction Projects, Technical University of Catalonia (UPC), c/ Colom 11, Ed. TR5, Terrassa (Barcelona) 08022, Spain; sergi.villalba@upc.edu

**Keywords:** fiber optics, structural health monitoring, distributed fiber sensing, time domain reflectometry, frequency domain reflectometry, civil engineering

## Abstract

The application of structural health monitoring (SHM) systems to civil engineering structures has been a developing studied and practiced topic, that has allowed for a better understanding of structures’ conditions and increasingly lead to a more cost-effective management of those infrastructures. In this field, the use of fiber optic sensors has been studied, discussed and practiced with encouraging results. The possibility of understanding and monitor the distributed behavior of extensive stretches of critical structures it’s an enormous advantage that distributed fiber optic sensing provides to SHM systems. In the past decade, several R & D studies have been performed with the goal of improving the knowledge and developing new techniques associated with the application of distributed optical fiber sensors (DOFS) in order to widen the range of applications of these sensors and also to obtain more correct and reliable data. This paper presents, after a brief introduction to the theoretical background of DOFS, the latest developments related with the improvement of these products by presenting a wide range of laboratory experiments as well as an extended review of their diverse applications in civil engineering structures.

## 1. Introduction

### 1.1. Motivation for SHM

The highway and railway transportation systems are considered to be some of society’s critical foundations. In this complex system, bridges and viaducts assume an important role because of their distinct function of joining these networks as their crucial nodes. They face continuously increasing traffic volumes and heavier truck and rail-loads, which degrade the long-term performance of these structures. In the United States alone, over 11% of the nation’s 607,380 bridges are deemed structurally deficient and the cost to repair these deficient bridges is estimated to be $76 billion [1]. Well maintained civil infrastructure can substantially increase a country’s competitiveness in a global economy and enhance resilience to adverse circumstances. Therefore, a structure, especially in the present days, must be able to reliably produce information regarding any alterations in its structural health condition and communicate it to the responsible operators and decision makers both in a timely way and either automatically or on-demand in order to decrease these costs. To achieve such goals, a modern structure needs to be prepared with a system that includes a “nervous subsystem”, a “brain”, and “voice lines”, which is continuously in operation and able to sense structural conditions [2]. 

The control and monitoring of the aging process of civil engineering structures is of extreme importance for their quality and safety. Furthermore, there are different external events that can damage a structure. Damage can be defined as alterations that when introduced into a system will have an adverse effect in its current or future performance [3]. The process of employing a damage identification strategy for engineering and aerospace infrastructures is referred to as structural health monitoring (SHM). When dealing with long-term SHM, the goal of the system is to deliver updated information regarding the ability of the structure to continue to serve its intended purpose and function despite the unavoidable effects of the mentioned aging process and the accumulation of damage that results from the operational environment. SHM systems are also able to rapidly assess and screen the structure condition under an accidental or extreme event such as earthquakes, increase of water levels or unexpected loadings. The early detection of structural malfunctions allows the increase of the service life-time of the structure at the same time that decreases the maintenance costs. 

The act of damage identification has been around probably, in a qualitative manner, since man started to using tools [3]. Notwithstanding, SHM has been recently a fast-developing area in aerospace and engineering disciplines especially in the civil engineering field. The innovation in the SHM technologies as well as the development of large-scale SHM systems has been a great subject of interest within the engineering and academic communities over the last two decades [4,5,6,7,8,9,10,11]. However, despite its great potential, SHM has not been applied in large scale and in a systematic manner to civil infrastructures. One significant reason for this is the lack of reliable and affordable generic monitoring solutions [12].

### 1.2. Optical Fiber Technology in SHM of Built Environment

Currently, assessment of buildings, bridges, dams, tunnels and other vital civil engineering infrastructures are carried out by engineers trained in visual inspection, which sometimes can be inaccurate due to differences in their background for safety condition assessment. In order to improve the inspection accuracy and efficiency, optical fiber sensors (OFS) are one of the fastest growing and most promising researched areas, due to their features of durability, stability, small size and insensitivity to external electromagnetic perturbations, which makes them ideal for the long-term health assessment of built environment [13].

Furthermore, the most regularly practiced SHM approaches are based on electric strain sensors, accelerometers, inclinometers, GNSS-based sensors, acoustic emission, wave propagation, *etc*. Nevertheless, all of them present genuine challenges when deployed in real world applications [4]. Different kinds of sensors, embedded or attached to the structure, can be used in SHM systems but only those based on fiber technology provide the ability to accomplish integrated, quasi-distributed, and truly distributed measurements on or even inside the structure, along extensive lengths [2].

Standard monitoring practice is normally based on the choice of a limited and relatively small number of points that are supposed to be illustrative of the structural behavior [14]. For a large scale structure, the number of point sensors needed to generate complete strain information can grow rapidly. Discrete short-gauge sensors provide useful and interesting data of the structure related with local behavior but might omit important information in locations where degradation occurs but that is not instrumented. There are ways of covering larger extensions of structures with the application of long-gauge sensors allowing the detection and characterization of phenomenon’s that have a global impact on the structure. Notwithstanding, the reliable detection and characterization that occurs far from instrumented areas continues to be challenging since it depends on high-level algorithms whose performance may decrease due to external interferences that can mask the real damage, such as temperature variations, load changes, outliers and missing data in monitoring results [15]. Distributed optical fiber sensors (DOFS) offer an advantage over point sensors for global strain measurements.

The thousands of sensing points that the DOFS provides enables mapping of strain distributions in two or even three dimensions. Thus, real measurements can be used to reveal the global behavior of a structure rather than extrapolation from a few point measurements. 

A truly distributed optical sensor is expected to measure temperature, strain and vibration data at any point along an entire fiber through light scattering. The great challenge has been to develop these sensors in a way that they can achieve appropriate sensitivity and spatial resolution [13]. Fortunately, great advances have been made in the last decades in order to improve this area.

Several general state of the art papers of FOS have been published [16,17,18,19] and also various where the applications of these sensors on civil engineering structures were reviewed [10,20,21,22]. A comprehensive DOFS state of the art paper was presented in [11] where the theoretical background of the most used DOFS techniques was extensively elaborated and where some civil engineering applications, especially focused on bridges, were presented. In the present paper, a broader and wider range of civil engineering applications are presented (geotechnical structures, pipelines, dams, bridges, laboratory experiments) as some other innovative implementations. Furthermore, and finally, a review of the dynamic capabilities of distributed sensing and the most practical challenges associated with the implementation of these sensors are presented.

## 2. Fiber Optic Sensors

### 2.1. Introduction

The first reference of fiber optic sensors relates to the flexible endoscopes developed in the first half of the twentieth century. With it came a revolution in the medicine field that continues to the present day [23]. However, the start of the development of modern age of optical fiber sensors started in earnest in 1977 for long distance telecommunications and it has experienced an exponential growth during the last four decades. Sensing applications are a small spin-off from this technology, taking advantage of developments in optoelectronic components and concepts. By 1982, magnetic, acoustic, pressure, temperature, acceleration, gyro, displacement, fluid level, torque, photo acoustic, current, and strain sensors were among the fiber optic sensors already developed and being researched [24] This modern age of fiber optic sensors was possible thanks to the development of extremely low-loss optical fibers in the late 1970s [23].

The fiber optic communications industry has literally revolutionized the telecommunications industry by providing higher-performance, more reliable telecommunication links with ever-decreasing bandwidth cost. As component prices have fallen and quality improvements have been made, the capability of fiber optic sensors to replace the more traditional electric sensors has been improved [25].

There are many inherent advantages related to the application of fiber optic sensors. Among these, it’s possible to highlight their resistance to electromagnetic interference, their light weight, small size, high sensitivity, high-temperature performance, immunity to corrosion and large bandwidth.

**Figure 1:**
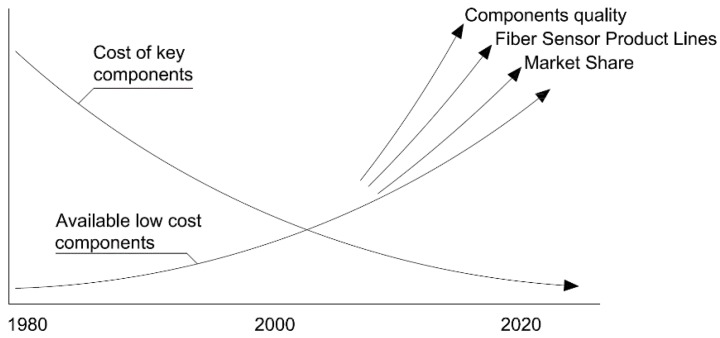
Trends for fiber optic sensors (adapted from [23]).

Initial introduction of this technology into the markets that were directly competing with conventional sensor technology in the last two decades of the past century was relatively slow. This was largely related to the high cost of suitable components. However, this situation has since changed and the projections for the future are tremendously optimistic as can be seen in Figure 1.

With the development of each new successful fiber optic product, the cost for existing and newly introduced components continues to drop. By the year 2020 there are many areas where is expected to occur a rapid growth of fiber optics sensors applications. From medical instrumentation, aerospace and industrial applications to structural health monitoring and damage assessment systems in civil structures, the ever-increasing capabilities and lower costs of this technology make it very attractive to end users [23].

Fiber optic sensor based monitoring methods are highly welcome for non-destructive assessment of all types of engineering structures mainly because of the following reasons: they can’t be destroyed by lightning strikes, can survive in chemically aggressive environments, they can be integrated into very tight areas of structural components, and finally are able to form sensor chains using a single fiber [26].

### 2.2. Basics of Fiber Optic Sensors

**Figure 2:**
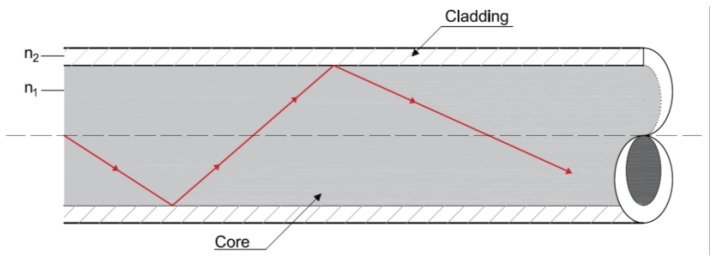
Light guiding and reflection in an optical fiber.

In a simple way, an optical fiber is a cylindrical symmetric structure that is composed by a central “core” with a diameter between 4 and 600 µm and a uniform refractive index [27]. It’s then enclosed by a “cladding” with a relative lower refractive index trapping the light waves being carried in the core by the reflection at the interface between core and cladding Figure 2. In order to provide environmental and mechanical protection to the fiber, this cladding can be covered with an external plastic coating.

Since the optical fiber is a physical medium it is constantly exposed to external perturbations. In this way, it experiences geometrical and optical changes due to those same perturbations. For communication applications it is preferred to minimize these effects in order to provide a reliable signal transmission and reception. However, in fiber optic sensing, the response to these external induced effects is intentionally enhanced [28]. This change of some of the properties of the guided light can be produced inside or outside (in another medium) of the optical fiber. Therefore, two different types of sensors can be differentiated: extrinsic and intrinsic [29].

**Figure 3:**
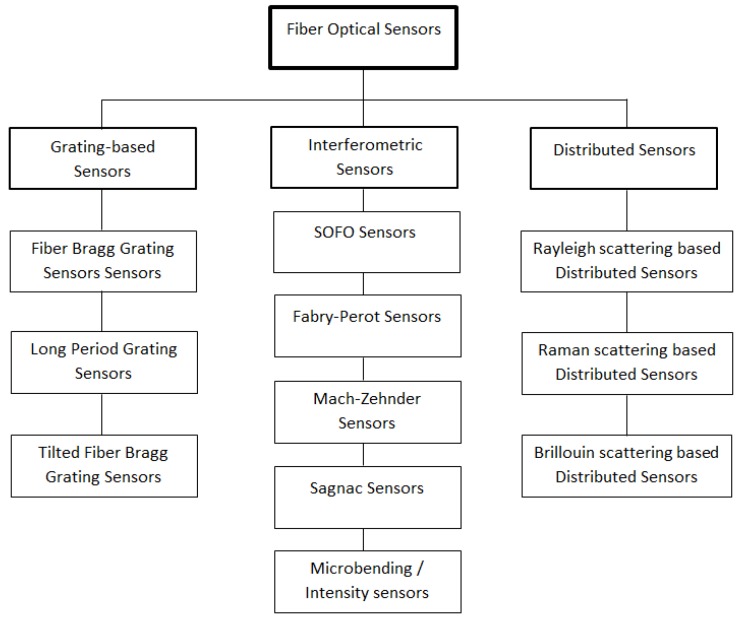
Overview of fiber optic sensor technologies (adapted from [30]).

In turn, each of these classes of fibers has various subclasses, and even in some cases, sub-subclasses that consist of a large number of fiber sensors. There are different ways to classify optical fiber sensors (OFS) depending of which property is being considered, *i.e.*, modulation and demodulation process, application, measurement points, *etc.* [28]. However, and taking into account the goal of this paper, they can be categorized into three different classes: interferometric sensors, grating-based sensors and distributed sensors [30] (Figure 3). The first two have been widely investigated and used in civil engineering monitoring applications [31,32,33,34,35,36]. Therefore, the interest of this paper is focused on the latter.

### 2.3. Distributed Fiber Optic Sensors

Distributed Optical Fiber Sensors (DOFS) share the same advantages of other OFS. However, they offer the possibility of monitoring variations of one-dimensional structural physical fields along the entire optical fiber in a truly distributed way. Furthermore, an additional benefit regarding distributed sensing is that it only requires a single connection cable in order to communicate the acquired data to the reading unit in opposite of the large number of otherwise required connecting cables when using discrete sensors. This characteristic makes DOFS more cost-effective and at the same time opens a wide range of important applications such as the continuous (in space and time) monitoring of large civil engineering structures.

It should be noted, that distributed sensing can, in a sense, also be achieved through the use of quasi-distributed FBG sensors. In addition, this has been the most popular optic fiber technique for spatial continuous measurements, since 2/3 of the SHM projects which includes fiber sensors, have opted for quasi-distributed FBG [21]. However, in this technique, instead of allowing a continuous monitoring along the fiber path, a finite number of locations are measured. The most effective and used type of quasi-distributed sensors are based on Fiber Bragg gratings by multiplexing many of these sensors in the wavelength domain [37]. Thus, FBG sensing relies on wavelength division multiplexing (WDM) to serve as a quasi-distributed sensor. Using this method usually limits the number of gratings that can be multiplexed to fewer than 100. However, by applying time division multiplexing (TDM) to each wavelength channel and by assigning one central wavelength for each grating an increase in the numbers that can be integrated by these sensors is achieved.

With DOFS technology, the fibers are bonded to the surface or embedded inside the material [2]. When strain and temperature changes are transferred to the optical fiber, the scattered signal within the fiber is modulated by these physical parameters. By measuring the variation of this modulated signal, distributed fiber sensing is achieved.

#### 2.3.1. Scattering in Optical Fibers

Scattering is at the origin of truly distributed fiber optic sensors (DOFS) and it can be defined, in a simple way, as the interaction between the light and an optical medium. Three different scattering processes may occur in a DOFS, namely: Raman, Brillouin and Rayleigh scattering [38].

When an electromagnetic wave is launched into an optical fiber, its propagation through the medium interacts with the constituent atoms and molecules and the electric field induces a time dependent polarization dipole. This induced dipole generates a secondary electromagnetic wave and this is called light scattering [13]. When the medium, where this scattering occurs, is homogenous only a forward scattered beam is allowed. However, the optical fiber is an inhomogeneous medium due to its variations in density and composition and in this way, by the interaction of the light beam with the fiber, some photons return toward the light source producing backscattering. This back propagating light can be then used to get information about the fiber properties and consequently of the environmental effects to which is subjected to.

Raman scattering is greatly dependent of the temperature of the fiber which has been explored in order to instrument very successful techniques with various applications in different areas [39,40,41] but also distributed temperature sensors used, for example in the detection of water leakage in dikes [42].

In a similar way, Brillouin scattering is intrinsically dependent on the fiber density, which in its turn is related with temperature and strain being in this way exploited in Brillouin based DOFS [43,44,45,46,47,48].

On the other hand, Rayleigh scattering, as a quasi-elastic or linear phenomenon, is by itself independent of almost any external physical field. In fact, in Rayleigh-based DOFSs the scattering is used to measure propagation effects, which can include attenuation and gain, phase interference and polarization variation. It’s worth mentioning that Raman and Brillouin scattering are also influenced by these propagation effects but since they present a direct relation to the measured parameters, these effects are usually neglected.

Distributed fiber optic sensors can depend on different techniques and principles. For different SHM applications, different DOFSs sensors can be developed and so different techniques are applied. In the next subsections, the most important techniques related to the scope of this document are described.

#### 2.3.2. Time Domain Reflectometry—OTDR

**Figure 4:**
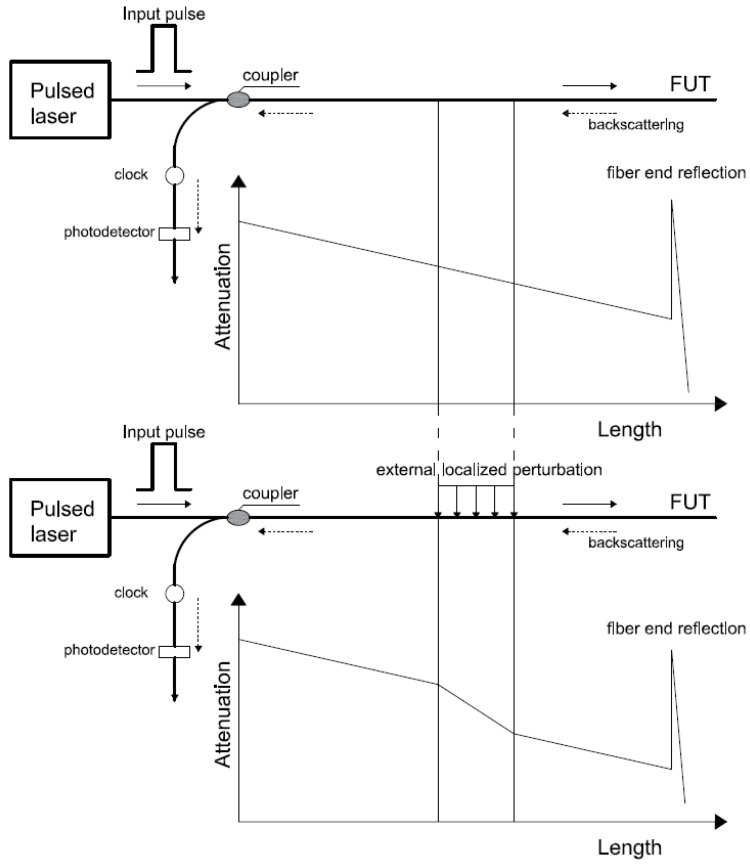
Principle OTDR based on Rayleigh backscattering.

Truly or intrinsic distributed sensing techniques emerged at the beginning of the 1980s when a technique called Optical Time-Domain Reflectometer (OTDR) was created for testing optical cables in the telecommunications industry. In OTDR technique, a short optical pulse is launched into the fiber and then a photo detector processes the amount of light being backscattered as the beam propagates along the fiber. In this process, loss occurs due to the Rayleigh scattering that is a result of the microscopic and random variations of the fiber core refractive index as mentioned before. By monitoring the variations of the intensity of the Rayleigh backscattered signal, the spatial variations in the fiber scattering coefficient are detected or, as it’s more commonly called, attenuation [25]. If the optical fiber is not affected by any external phenomenon, this attenuation decays exponentially with time. On the other hand, when the optical fiber under test (FUT) is subject to an external perturbation, attenuation will present a sudden variation localized on the perturbation as shown in Figure 4.

The spatial resolution of an OTDR instrument is the smallest distance between two scatters that can be resolved. For c standing for the velocity of light, n for the refractive index of the fiber and τ for the pulse width, the spatial resolution is given by Equation (1):

(1) $$\Delta {\text{Z}}_{\text{min}}=\frac{c\tau }{2n}$$

For a OTDR instrument with a pulse width of 10 ns, a current fiber refractive index of n=1.5 and the velocity of light as $c=3\times 10^{8}\;\text{m}/s$, from Equation (1) we obtain a spatial resolution of ≈1 m, which is relatively low, limiting the possible applications of OTDR in SHM. In order to increase this spatial resolution, the pulse width has to be reduced. However, in doing so, a decrease in the launched pulse energy may occur, also decreasing the detected light signal, and in this way, weakening the signal-to-noise ratio. This is problematic, especially for long-range sensing applications, so a basic tradeoff between the spatial resolution and the sensing range is inevitable, requiring the optimization of the pulse width and pulse energy based on each application requirements [25].

As mentioned before, Raman and Brillouin scattering have been also used for distributing sensing applications. OTDR based on Raman scattering was developed to sense the ambient temperature during the 1980s.

##### BOTDR and BOTDA

**Figure 5:**
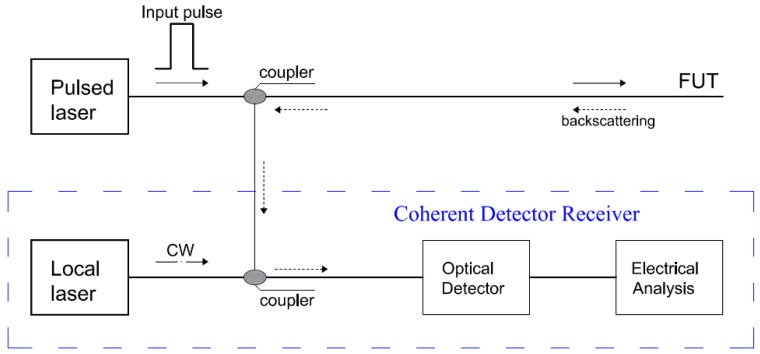
Typical configuration of a BOTDR setup.

As mentioned above, Brillouin scattering occurs from acoustic vibrations stimulated in the optical fiber [49]. These vibrations produce a counterpropagating wave, called Brillouin scattering wave, which weakens the forward-moving input pulse. To satisfy the requirement of energy conservation, there is a frequency shift between the Brillouin scattering wave and the original light pulse frequency. This frequency varies for different temperature and longitudinal strain conditions, making it possible to measure strain and temperature distribution based on the Brillouin scattering effect [50]. Brillouin optical time domain reflectometer (BOTDR, Figure 5), is based on the spontaneous Brillouin scattering and was initially introduced as a way to enhance the range of OTDR and with the advantage of monitoring the system from one end of the sensing fiber [51]. Brillouin optical time domain reflectometer (BOTDR) sensors thus mark the beginning of strain and temperature measuring DOFSs. 

**Figure 6:**
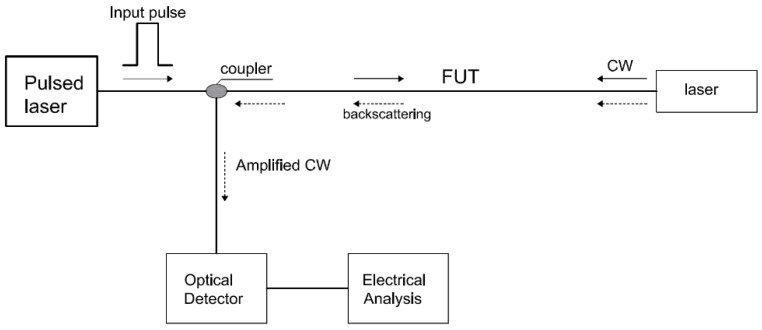
Typical configuration of a BOTDA setup.

On the other hand, Brillouin optical time domain analysis (BOTDA, Figure 6), is based on stimulated Brillouin scattering. This technique uses two counter propagating lasers and takes advantage of Brillouin amplification [13].

A coherent detection technique is necessary to detect the usually very weak counterpropagating Brillouin scattering signal [52]. In this way, the fiber is interrogated with a continuous wave (CW) from one end. Stimulation of the Brillouin scattering process occurs when the frequency difference of the CW signal and the pulse is equal to the Brillouin shift (resonance condition) [22]. 

The first early works on Brillouin-based DOFS were focused on temperature sensors. Among those works, [43,44,53] can be highlighted. The latter investigated the design of an optimized distributed sensor for temperature measurements based on the Brillouin gain/loss mechanism presenting data from a 51 km system, which was at that date, the longest sensing length reported.

BOTDR is normally capable of long-distance distributed sensing with a sensitivity of 5 µε, which is suited for large-scale applications of structural and geotechnical monitoring [54]. However, both BOTDR and BOTDA are limited to a spatial resolution of roughly 1 m and therefore not suitable for a large range of structural monitoring applications. Different techniques have been studied with the intention of improving said resolution [55], such as the Brillouin optical correlation domain analysis (BOCDA) that improves this resolution to the cm level [56,57,58]. Furthermore, complex and advanced algorithms were studied and developed for several Brillouin backscattering based DOFS applications with the objective of improving its spatial resolution [59,60]. Notwithstanding, Brillouin backscattering based DOFS constitute the most studied and used DOFS systems in civil engineering structure SHM applications.

#### 2.3.3. Frequency Domain Reflectometry

The goal of short spatial resolutions of millimeter scale and cost effective distributed fiber sensors has increased interest in Optical Frequency Domain Reflectometry (OFDR) systems [61,62,63]. In order to obtain a high spatial resolution with OTDR-based sensors, a very narrow light pulse is required, resulting in a proportionally lower level of the backscattering signal and, at the same time, an increased receiver bandwidth requirement in order to detect such signals [25]. Hence, the noise level is also expected to increase making the detection of small variations in the backscattered signal due to strain and temperature almost impossible. These combined factors make OTDR-based DOFSs with high spatial resolution very expensive systems. In this way, a DOFS with millimeter scale spatial resolution has been developed based on Rayleigh scattering OFDR [64,65,66]. This is the so called Optical Backscattered Reflectometer (OBR).

Rayleigh scattering originates from the interaction between the electromagnetic wave propagating in the fiber core and silica impurities [42]. In a simple way, the Rayleigh backscatter profile of a determined optical fiber results from its heterogeneous reflective index, which is distributed randomly along the whole length of the fiber. This constitutes as a fingerprint of each optical fiber and is a result of its manufacturing process [67]. When the fiber is subjected to an external stimulus (like strain) the backscatter pattern presents a spectral shift that is then used to calculate the strain changes along the length of the fiber by comparing it with the unaltered (unstrained) reference state.

Instead of reading the intensity of the Rayleigh backscattered signal, OFDR measures the interference fringes of the Rayleigh scattered light from a tunable laser source and a static reference fiber in frequency domain. By means of the inverse Fourier transformation, the amplitude and phase in frequency domain are converted to the time/spatial domain [22].

Froggatt and Moore described the random fluctuation of the index of refraction that causes Rayleigh scattering as an equivalent FBG [66]. From this perspective, the whole length of the optical fiber is divided in several short sections (in the orders of centimeters) that are equivalent as a weak random FBG with a Swept-wavelength Interferometry (SWI). While the fiber is not perturbed, while random, this equivalent weak FBG is stable in time.

By scanning the frequency with the OFDR technique the spectral response of each equivalent FBG is obtained and in this way, strain and temperature variations with high spatial resolution. This resolution (Δz) is related to the optical frequency sweep range of the tunable laser source (ΔF) as given by Equation (2) where c is the speed of light and n the fiber refractive index:

(2) $$\Delta \text{Z}=\frac{c}{2n\Delta F}$$

The OBR-based DOFSs are excellent for short sensing lengths (<100 m [68,69,70]). Nonetheless, longer sensing systems are possible at the cost of spatial resolution, and temperature/strain resolution. Koshikiya *et al.*, for instance, reported the detection of high loss points with cm level resolution over 5 km measurement range with high sensitivity and a noise level 23 dB lower than the Rayleigh backscatter [71].

Notwithstanding, the OBR technique, with its high spatial resolution, even if limited to some hundred meters, addresses some applications that aren’t easily covered either by Brillouin- or by Raman-based DOFSs [72].

A review of the performance of the discussed distributed sensing techniques as reported by different research studies is presented below in Table 1. The multiplexed FBG sensor technique is also included for comparison.

As already mentioned, Brillouin-based DOFSs are currently the most studied and applied measuring systems in civil structure SHM. This is due to their extended measurement range potential that makes them very useful for application on large structures, such as dams, pipelines, tunnels and long span bridges. Notwithstanding, some applications require a better spatial resolution than that provided by these sensors. The BOTDA sensing technique, through the application of advanced and complex algorithms, can address this point but in the process increases the price of this technology. The OBR technique (Rayleigh OFDR) offers a more cost-effective way of achieving high spatial resolution at the cost of limiting, nonetheless, the sensing range to about 70 m. 

## 3. Civil Engineering SHM Applications with DOFS

The great majority of photonic sensing technology applied in the civil engineering area is constituted by discrete sensors such as FBG. This topic has been extensively discussed in different publications in the past decades [20,77,78,79,80,81,82]. Taking in account the scope of this state-of-the-art paper, only applications of truly distributed sensing with fiber optics technology are presented.

Nowadays, DOFS sensors are an attractive technology that offers superior performance and advantages when compared with more conventional sensors applied in SHM practice. Despite their apparently high cost, they are ideal for applications where reliability in challenging environments is essential. Furthermore, they offer lower installation and maintenance costs.

However, this is still a recent and developing technology as it can be perceived by the relatively few number of DOFS applications in SHM projects. Notwithstanding, some different DOFSs applications were made in the last two decades in different civil engineering structures such as bridges, dams, tunnels, pipelines and slopes that are presented below. Also, a great amount of work has been done, and discussed below, with the goal of improving these sensors’ capabilities by executing different laboratory experiments.

### 3.1. Laboratory Tests

In order to assure the proper operation of DOFS, one of the most important aspects to consider is the correct transfer of the measurands from the monitored structure to the sensor. Zeng *et al.*, for example, performed a strain measurement of a 1.65 m reinforced concrete beam with a Brillouin scatter-based DOFS where two different installation methods were analyzed: fiber embedded in glass fiber reinforced polymer—GFRP—rods and fiber bonded to steel reinforcing bars [45]. Both methods were found to effectively protect the optical fiber strand and measure strain data. On the other hand, Hoult *et al.* performed a series of axial tension tests on steel plates using polyimide and nylon-coated sensing cables [83]. Overall, the polyimide-coated fiber presented a higher accuracy and a better correlation with the results obtained with electrical strain gauges. However, its measurements seemed erroneous at crack locations. On the other hand, the nylon-coated fiber, although it can be used for crack detection and general deterioration, doesn’t offer the same accuracy as the polyimide cable due to the slippage between the nylon coating and inner core.

**Figure 7:**
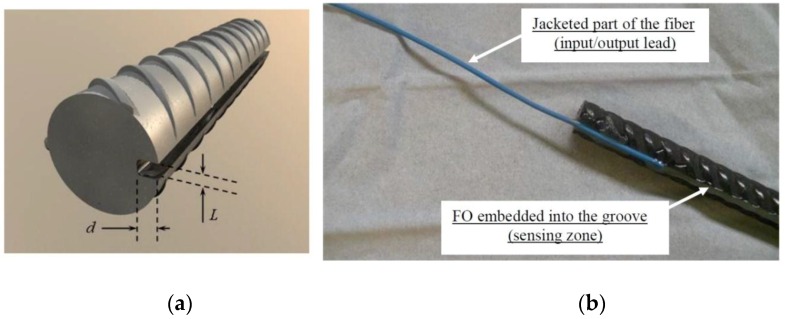
Geometry of the groove in the rebar (**a**) detail view of the end of the bonded zone (**b**) [84].

Furthermore, sometimes, the DOFS protection can interfere in the collection of correct strain measurements. In order to overcome, or at least reduce these problems, and in this way effectively measure the strain of a RC structure after cracking, Quiertant *et al.* tested through a series of experiments the implementation of an OBR interrogation unit paired with fiber optic sensors in rebars with the goal of obtaining strain measurements in reinforced concrete structures made of Ultra High Performance Fiber Reinforced Concrete as seen in Figure 7 [84].

The tested parameters of these experiments were the chosen method for the installation of the optical fiber, *i.e.*, an FO bonded on the rebar surface or mounted into a groove, the geometry of the groove and finally the typology of the used optical fiber (subject in its majority to its coating material, ~250 µm diameter polyimide or ~190 µm acrylate coated fiber). It was then found that a fiber optic sensor with a polyimide coating embedded in a groove cut along the rebar presented the best results.

**Figure 8:**
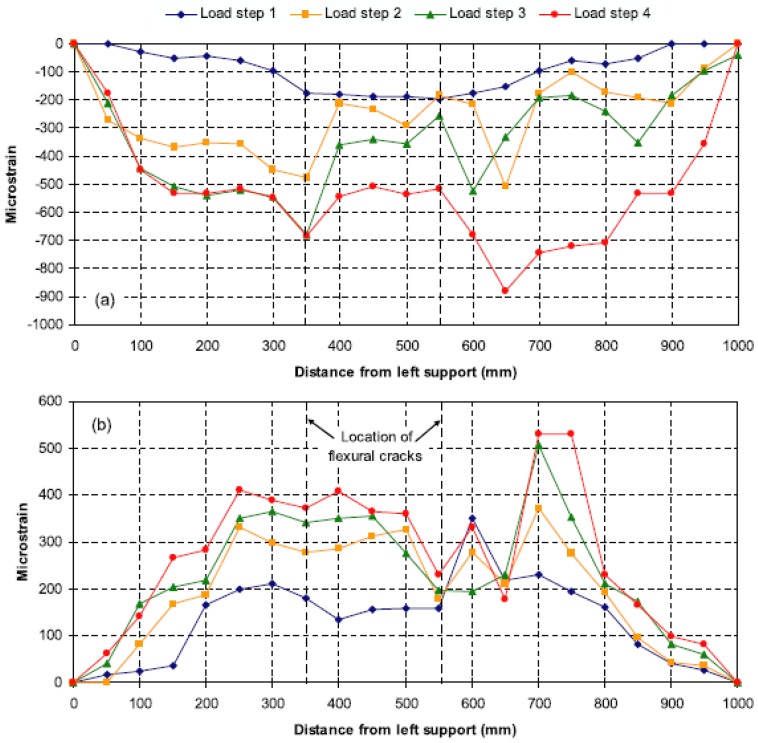
(**a**) Compressive and (**b**) tensile strain distributions measured by distributed Brillouin sensors [85].

As already mentioned, one of the most sought-out applications for DOFS is the crack detection in RC structures. However, one of the limitations of Brillouin-based DOFS is related to the fact that very significant strain changes that occur over lengths shorter than one-half of the spatial resolution are not detected and measured, making the application of this technology to crack detection very difficult. Deif *et al.* studied the possibility of enhancing the spatial and strain resolutions of distributed BOTDA sensors in order to overcome this issue [85]. A multiple-peak fitting method was applied to extract the strain distributions on the top and bottom surfaces along a RC beam that was subject to a four-point bending configuration. The multi-peak fitting method allowed the spatial resolution of the measurements to be improved from 15 cm (of the sensor) to 5 cm (read-out resolution). However, despite being able to capture damage build-up, the system was unable to detect and precisely locate the formation of cracks, as seen in Figure 8.

Glisic and Inaudi also evaluated the performance of the application of an advanced algorithm that was developed to avoid this limitation through several laboratory tests where the distributed temperature and strain monitoring system was based on the changes in Brillouin frequency [14]. For these tests a specific set-up was built and used to tension 10 cm of optical fiber with different previously defined values. All of these simulated crack openings were correctly and successfully detected and localized.

Furthermore, these authors, taking into account the risk of optical fiber breakage when the stress generated by the crack is very high and the fact that this developed algorithm was only suitable if local stress was redistributed over a minimum length of 10 cm, decided to create, at the location of the crack, a mechanism of sensor delamination to ensure that the system would continue to perform even for high strain levels. For this, they paid special attention to the selected adhesive.

This delamination mechanism and selected adhesive were then tested in laboratory with the use of a specific set-up where the DOFS was glued to metallic supports that were given a relative translation movement with the objective of simulating a 0.5 mm crack opening. The obtained test results proved the delamination mechanism and confirmed the good selection of the adhesive and the performed installation procedures. This demonstrated the capability of the system in detecting and localizing cracks with openings smaller than 0.5 mm. This system was later tested on a pipeline [86] and implemented in the monitoring of a bridge [14,87] with good performance of the implemented crack detection method.

**Figure 9:**
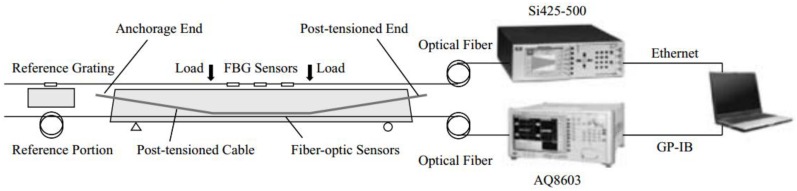
Configuration of the proposed SHM [88].

Zhang *et al.* investigated the development of a health monitoring system (HMS, Figure 9), that combined BOTDR and multiplexed FBG techniques with the intention of implementing it on rehabilitated concrete girder bridges [88]. For this, a series of static and dynamic loading tests to a simply supported reinforced concrete T-beam strengthened by externally post-tensioned tendons were executed.

This developed SHM system proved its great efficiency for operation by correctly identifying the relevant structural state for each loading state.

With the goal of exploring the capabilities of crack detection in concrete elements, without the occurrence of sensor failure and debonding, Villalba and Casas instrumented a concrete slab with OBR-based DOFS, glued to the bottom and upper surfaces, that were subject to a load test [89]. The slab was also instrumented with more conventional electrical sensors, such as embedded strain gauges and LVDTs for comparison purposes. A coating of polymer (polyimide) was used to protect the fiber. This technology was not only able to detect crack openings locations, but also to continue to correctly perform measurements up to load levels producing a crack width in the range of 1 mm. The results obtained in this experiment compared very well with the available data from the other instrumented sensors as well with visual inspection and predicted values of nonlinear FE models [90].

**Figure 10:**
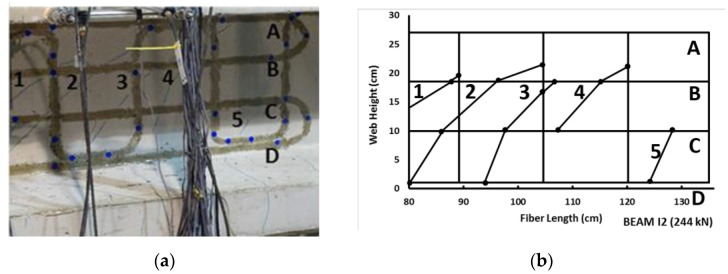
Comparison of real shear crack pattern (**a**) and obtained by OBR system (**b**) (adapted from [91]).

This group also implemented a novel technique in partially prestressed concrete (PPC) beams with OBR sensors in order to detect induced shear cracks. This is of extreme relevance since that contrary to what happens with bending cracking, where the cracks appear orthogonally to the beam axis, in the case of shear action, the inclination of the cracking pattern is previously unknown and may even change depending on the prestressing force and the location along the element [91]. For this, a two-dimensional DOFS grid was proposed that monitored the crack initiation, location, inclination and evolution during a beam load test, as seen in Figure 10.

These results showed good agreement with the visualized cracks and were obtained without requiring prior knowledge of the cracked zone and consequently proved the feasibility of this methodology.

Henault *et al.* conducted laboratory experiments where a concrete slab was instrumented with Raman-, Brillouin- and Rayleigh-based DOFS in order to obtain strain measurements (the Raman sensor was used for temperature compensation). BOTDR sensor results were limited by their poor accuracy (1 m spatial resolution combined with 20 µm/m) while OBR sensors provided very promising results [42]. However, since the applied load induced relatively small strains, temperature variations caused misinterpretations. The implemented DOFS were embedded in the concrete and the influence of the coating and installation process were also studied and highlighted.

**Figure 11:**
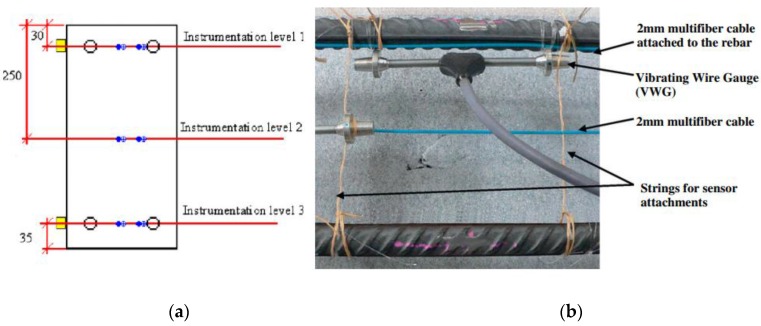
(**a**) Details of the cross-sectional instrumentation at mid-span of the beam (dimensions in mm); (**b**) Picture of the instrumented beam before concrete pouring (adapted from [92]).

The same research group also monitored a 3.4 m long reinforced concrete beam with a 0.25 × 0.5 m^2^ rectangular cross section that was subjected to a four-point bending test through the utilization of different Rayleigh scattering-based DOFS (three levels embedded in the concrete and two levels bonded to the external surface of the RC element) that were paired with Vibrating Wire Gauges (VWG) sensors as seen in Figure 11.

**Figure 12:**
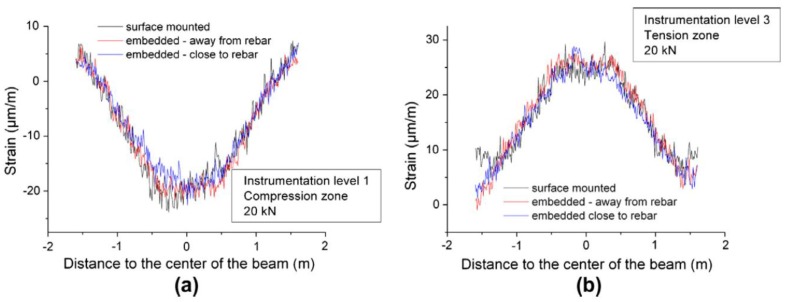
Strain profiles provided by DOFS system for a 20 kN bending load: (**a**) In the compression zone at instrumentation level 1 and (**b**) In the tension zone at instrumentation level 3 [92].

The test was divided in two phases. In the first one, the applied load level was below the concrete tensile resistance in order to check the performance of the different applied sensing systems. One interesting conclusion obtained from this part of the test was the much reduced influence of the location of sensors for strain measurement since the results obtained for the embedded (both close and far from the rebar) and the external bonded sensors presented a relatively good agreement both in compression and in tension, as seen in Figure 12.

**Figure 13:**
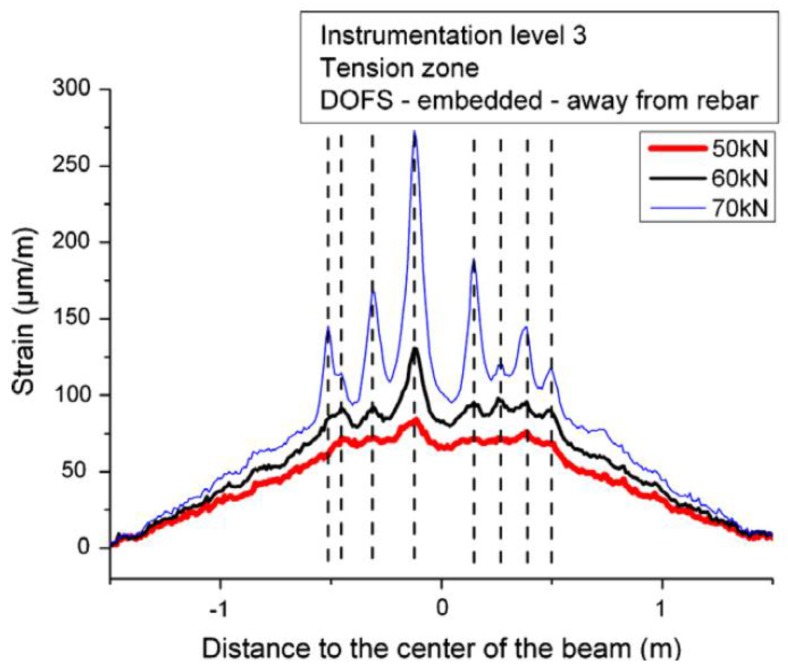
Tensile strain profiles recorded by the Rayleigh scattering based DOFS for various bending loads above 50 kN (adapted from [92]).

In the second phase, the incipient cracks were induced in order to assess the capability of the used DOFS sensors in detecting their location. Despite the fact that the RC specimen was severely damaged at high-load levels, the optical fiber cables didn’t present any tensile rupture. As seen in Figure 13, it was possible to detect the location of cracks (even before visual inspection) from the experimental data provided by DOFS and track its evolution.

The same investigation group developed an algorithm to automatically analyze crack evolution in RC elements [93]. This process provided a precise map of cracks with the evolution of their amplitudes and was tested with a four-point bending test like the one mentioned above.

**Figure 14:**
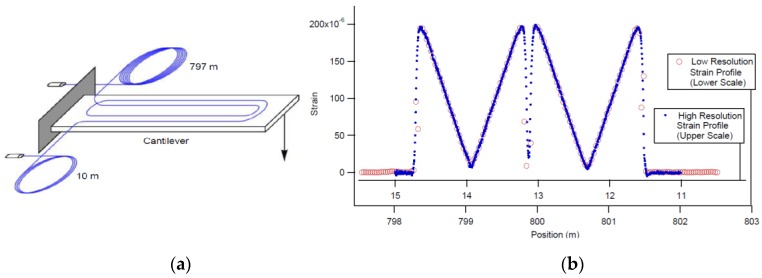
(**a**) Diagram of cantilever beam used for strain measurements. The fiber was adhered to the beam in a double loop and the two ends were spliced to leads of fiber with 10 m and 797 m lengths respectively; (**b**) Strain on the cantilever as measured from both fiber inputs (adapted from [95]).

One of the referred limitations of Rayleigh-OFDR-based sensing it’s the relatively short sensing range in order to obtain high spatial resolution measurements. However, Gifford *et al.* reported high precision measurements with OBR system over a two kilometer extension [94]. The same authors also successfully assessed the strain measurements capabilities of this test for both near (10 m) and long distances (≈1 km) through an experiment carried out on a cantilever beam as shown in Figure 14.

**Figure 15:**
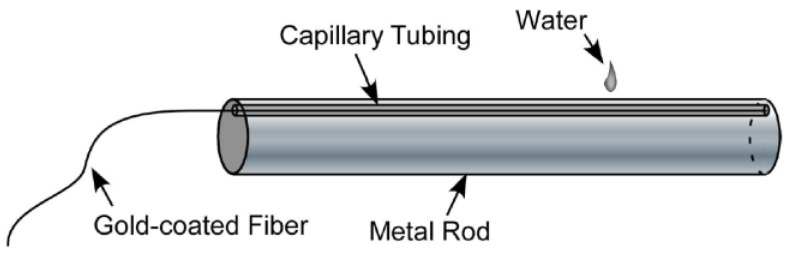
Test article for measuring at high temperatures with a strong thermal gradient [95].

This research group also performed another general test in order to validate the applicability of Rayleigh-OFDR sensors for temperature sensing [95]. This experiment consisted on a resistively heated metal rod that reached temperatures as high as 600 °C and then was subjected to a thermal gradient by dropping water on a localized spot on the rod that was instrumented with gold-coated optical fibers (to ensure fiber integrity at high temperatures) that were isolated in order to be unresponsive to strain Figure 15. 

**Figure 16:**
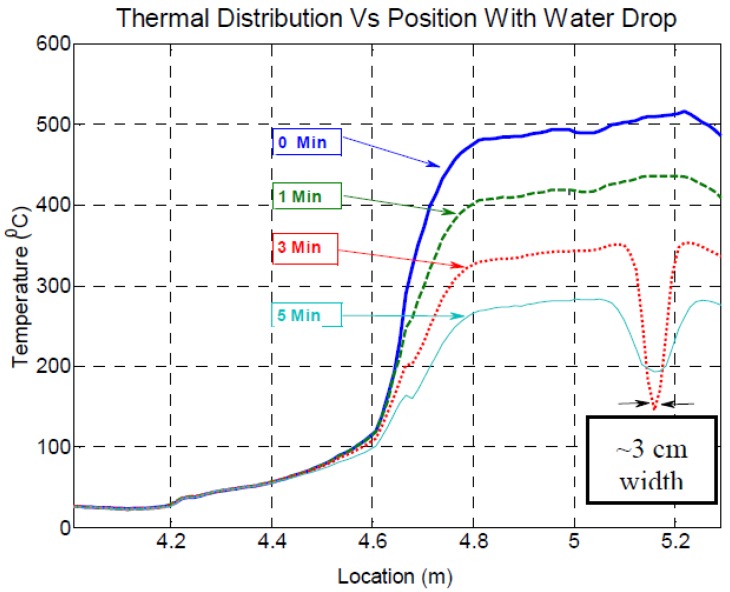
Temperature *vs.* location for fiber in heated metal rod (the fiber enters the rod at about 4.8 m) [95].

The results, as shown in Figure 16, confirmed the ability to measure temperature with a high spatial resolution with Rayleigh scatter-based DOFS.

**Figure 17:**
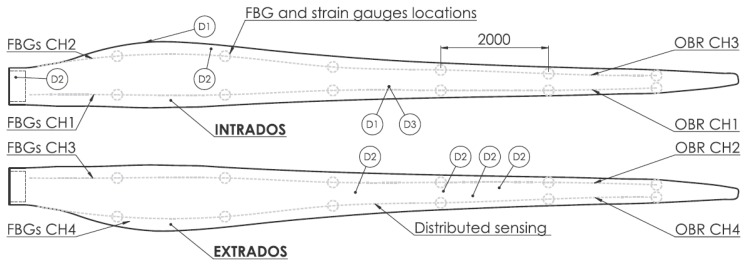
Sensors and damage locations in wind turbine blade. All units in mm [98].

Rayleigh backscatter-based DOFS were also reported in an application where localized heating was measured with millimeter spatial resolution [96]. Another successful application of Rayleigh based sensors for temperature measurements is reported by Sang *et al.* [97] in a nuclear reactor. More recently, Sierra-Pérez *et al.* compared the strain measurements of Fiber Bragg Gratings, and OBR-based distributed optical fiber and conventional electrical strain gauges sensors [98]. The two fiber optic technologies were deployed into a 13.5 m wind turbine blade subject to a load test. Several strain gauges were installed at the same locations than the FBGs and in additional places across the structure, as seen in Figure 17. 

**Figure 18:**
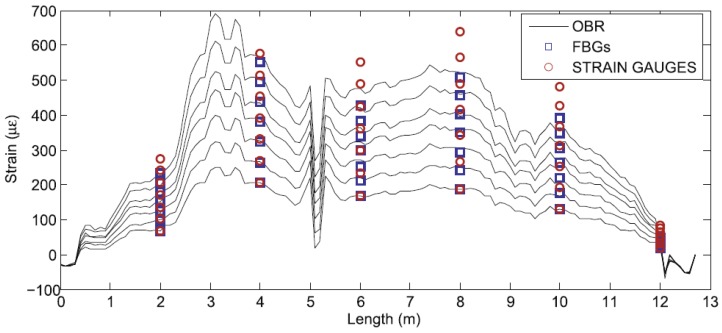
Example of strain profiles for seven load magnitudes gathered with the OBR, the FBGs and the strain gauges [98].

The fiber optics sensors proved to effectively measure strain and correspondent cracks induced during the test as seen in Figure 18.

### 3.2. Geotechnical Structures

**Figure 19:**
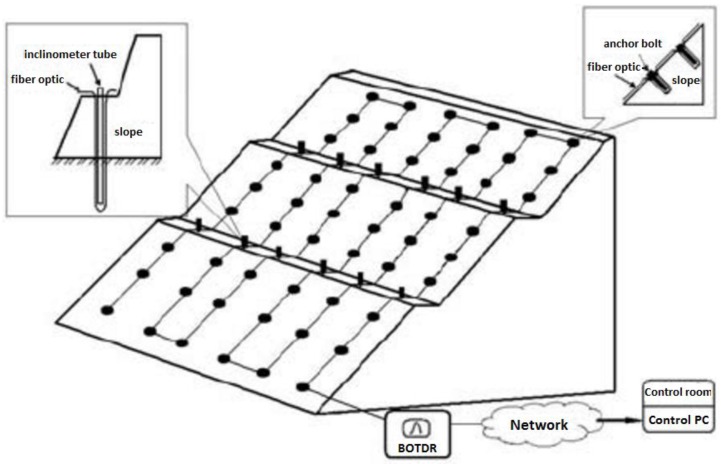
The diagram of BOTDR-based distributed fiber optical sensing monitoring system for slopes [99].

Due to its wide range capabilities, DOFS are becoming a very attractive technology for the instrumentation of geotechnical structures. However, when monitoring slope stability, the most common applied sensors are discrete ones such as inclinometers, crack detectors, reinforcement stress detectors and displacement meters. In this way, a global behavior of the slope is not easily obtained. To make things worse, these instruments are often incompatible with the deformation of the rock-soil mass, and the difficulties associated with installation procedure and bad measurements situations regularly turns the application of these sensors pointless. Shi *et al.* tested the feasibility of the application of a BOTDR-based distributed optical fiber sensing system for slopes as seen in Figure 19 [99]. 

**Figure 20:**
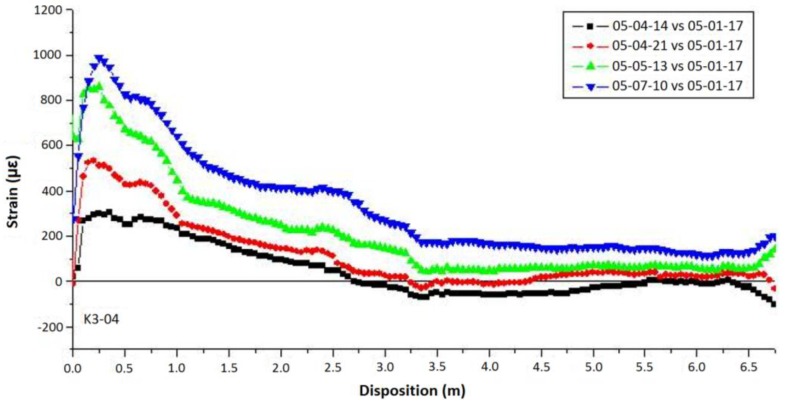
The time history plot of strain distribution along the K3-04 anchor axis [99].

The results are shown in Figure 20 for the period between January and July of 2005 where it’s possible to see the increase of strain, especially near the top of the anchor. This trend was related with the slope creep associated with the rainy season.

**Figure 21:**
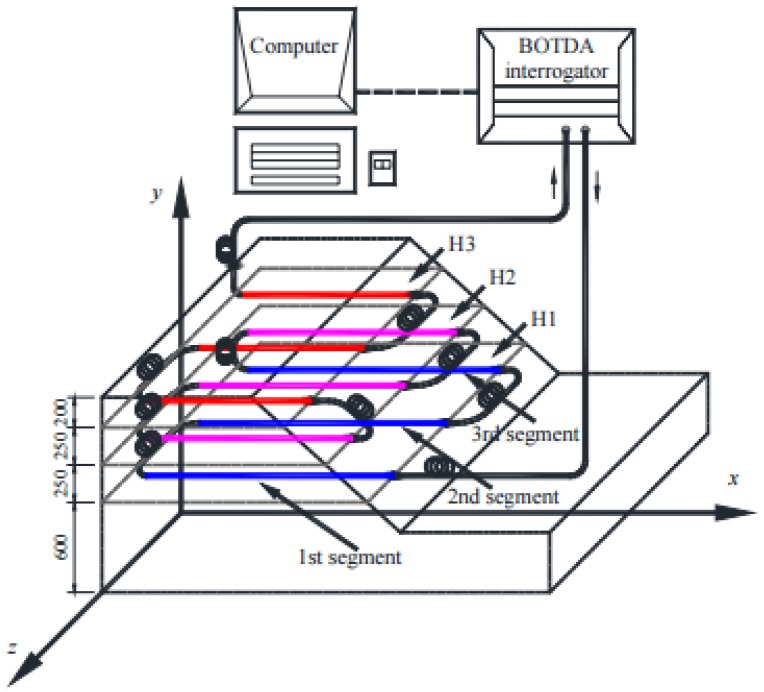
Layout of the BOTDA sensing fiber in the model slope (unit: mm) [100].

More recently, Zhu *et al.* built a medium-sized model of soil nailed slope in laboratory in order to assess the efficiency of monitoring slope stability problems through the employment of BOTDA sensing technology [100] (Figure 21).

The model was subjected to a surcharge loading test. Its results proved the feasibility of measuring horizontal strain distributions within the slope mass that reflect the deformation pattern that can be used to pinpoint the potential slip surface in the slope. Furthermore, it was concluded that the results obtained by the distributed fiber optic system can be used to estimate the stability conditions of the slope due to an empirical relationship between the characteristic maximum strain and factor of safety of the slope.

**Figure 22:**
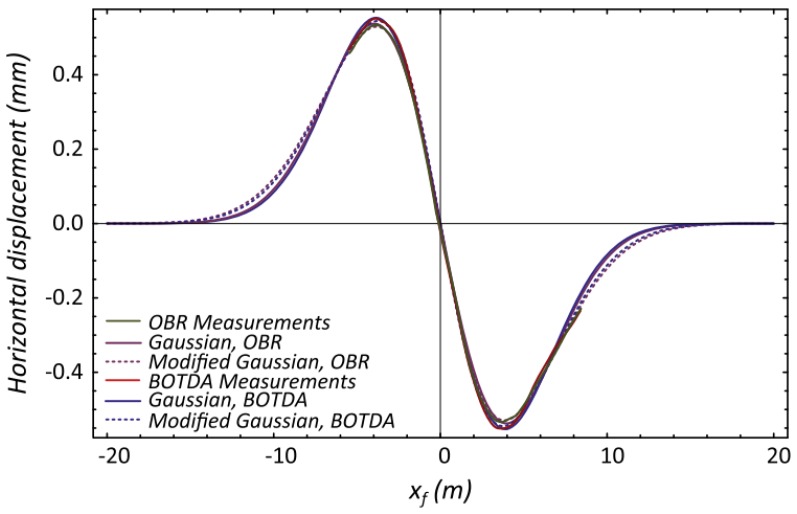
Integrated strain by OBR and BOTDA and best fit model for horizontal displacement from one of the field investigations [101].

Klar *et al.* assessed both BOTDA- and Rayleigh scattering-based DOFS capabilities of monitoring tunneling induced ground displacements [101]. This was based on the idea that a distributed optical fiber within the ground would deform due to the mentioned ground displacements. A detailed study of the soil-fiber interaction was presented by Klar and Linker where it was concluded that the DOFS essentially follows the same displacement of the soil due to its low rigidity [102]. This approach was tested in two different field investigations and it was concluded that both distributed fiber optic technologies provided results that could only be matched by multiple sub-millimeter displacement measurements and are more sensitive than laser-based displacement measurements (Figure 22). However, the authors pointed out the advantages of the OBR system due to its high-spatial resolution and possibility of conducting immediate measurements (less than 5 s) as opposed to BOTDA sensors (10 min).

The feasibility of applying the BOTDR technology for strain measurement in the Taiwan Strait Tunnel project was tested on the Nanjing Gulou tunnel (Nanjing, China) with good results as reported by Shi *et al.* [103]. This technology was also deployed for the strain measurement of the Xuanwuhu Lake tunnel in Nanjing, China [104], and Royal Mail tunnel at London (UK) [105]. 

In the framework of a system to detect sinkholes and embedded soil cavities, Lanticq *et al.* compared an OFDR-based strain sensor with a Brillouin-based one [106]. Tests performed on a railway tunnel suggested that the OFDR-based system is more effective owing to its better spatial resolution [72].

**Figure 23:**
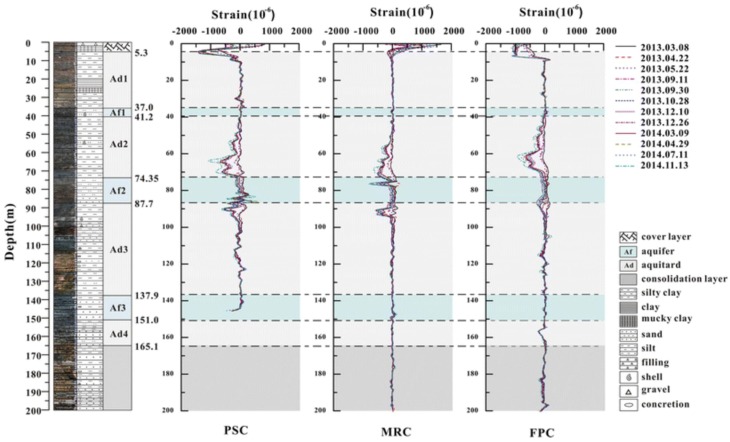
Drill core profile and strain distributions of three cables within 200 m depth [107].

Finally, Wu *et al.* applied BOTDR and FBG sensors in order to monitor the deformation of soil layers and pore water pressure in a 200 m borehole over nearly two years in Suzhou, China [107]. Three different types sensing fibers: polyurethane sheath cable (PSC), metal-reinforced cable (MRC) and 10 m fixed-point cable (FPC) were used, as seen in Figure 23. 

The results showed the incomparable potential of DOFS for soil subsidence monitoring as the strains at any depth of the soil layers were achieved. Regarding the different types of used cables, it was concluded that PSC was unable to monitor land subsidence at deep levels due to its small stiffness. Another borehole strata deformation monitoring with BOTDR-based sensors is also reported in [108].

### 3.3. Pipelines

**Figure 24:**
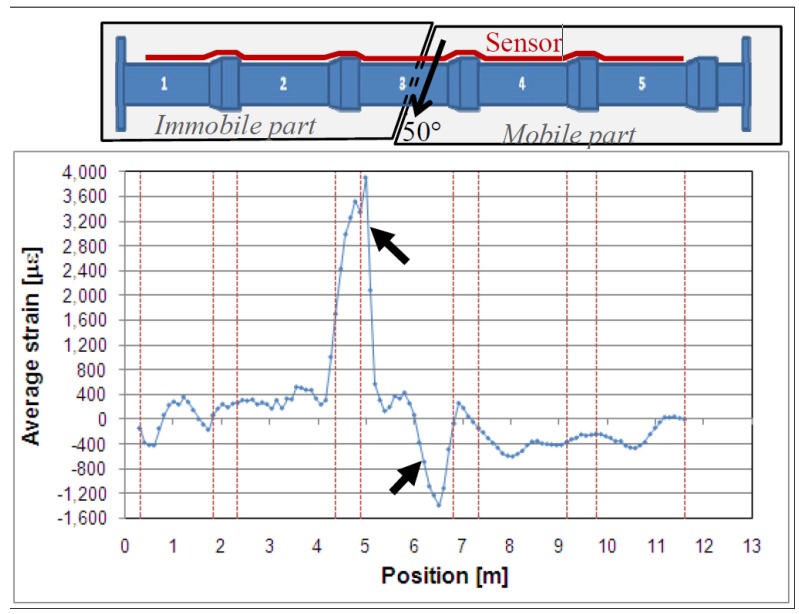
Results of test with detected damage (indicated by arrows) [109].

The application of distributed sensing technology in the Oil and Gas industry is of great interest and therefore has seen a substantially increase in recent years. Distributed and quasi-distributed techniques based on Brillouin and FBG technologies, respectively, have been the most commonly used [2]. Glisic and Yao performed a large scale test on a pipeline [86]. The main goal of this experiment was to develop a method for the implementation of a distributed fiber-optic system and at the same time provide a reliable way of real-time monitoring of pipelines that were subject to permanent ground displacements induced by earthquakes. In this way, a 13 m long concrete pipe with an exterior diameter of 30.48 cm was instrumented with a distributed fiber sensor based on Stimulated Brillouin scattering, along its length as shown in Figure 24.

In this test, a relative translation with an angle of approximately 50° was induced by hydraulic jacks between two parts, an immobile and a mobile part, Figure 24, where it is possible to observe a high tension point near joint number 2 and a high compression one close to joint number 3. These strain changes coincided with locations of damage in the pipeline. These proved the ability of the used system to detect cracks in close-to-real conditions and in real-time [14]. Another successful pipeline Brillouin DOFS monitoring example on a 35 years old gas pipeline near Rimini, Italy is described by Inaudi and Glisic [110]. In addition, Lim *et al.* 2015 were able to monitor the deformation of the cross section of a non-circular PVC pipe due to the dead weight of its carrying water reflected as oscillations in strain measurements made by BOTDA sensors that were deployed helically on the pipe [111].

### 3.4. Bridges

**Figure 25:**
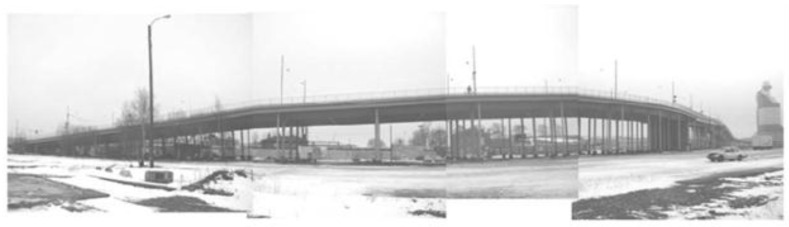
Götaälv bridge in Sweden [114].

One of the most appealing areas for the application of DOFS has been naturally the instrumentation of bridges. The Götaälv bridge (Figure 25) in Gothenburg (Sweden), is a 1000 m long bridge composed by a concrete slab poured on nine steel girders that are then supported on more than 50 columns [14,112,113]. The responsible traffic authorities, after the detection of several cracks in the steel girders required a continuous monitoring system for this structure. The capabilities of DOFS proved unique here since there was a great necessity of covering the full length of the structure as a crack could appear virtually at any point or in any girder. Hence, a DOFS system based on Stimulated Brillouin Scattering was successfully implemented and tested in 2007–2009 followed by a one year trial period [47,114]. Currently, the SHM of this bridge is still ongoing, making it probably the only true long-term implementation of DOFS on a real structure.

Another slab-on-girder bridge monitoring with a Brillouin based DOFS is reported in Matta *et al.* [115]. A 1.16 km optical circuit was implemented onto the girders for strain measurement and temperature compensation. The overall response of the girder was successfully measured by the BOTDR system and verified by a high-precision total station system.

A complete and interesting long term monitoring application is described by Glisic *et al.* in [116]. Here DOFS were embedded in the concrete during construction of the Streicker Bridge (a pedestrian bridge located on the Princeton University campus) allowing for the acquisition of important data related with the global performance of the structure, *i.e.*, from the early behavior of the concrete to the identification of damage, which was unprecedented. These sensors were validated by FBG sensors that were also deployed on this structure.

Bastianini *et al.* compared two different Brillouin sensor installation techniques, near-to-surface fiber (NSF) embedding and “smart” FRP sensor bonding [117]. The two systems were implemented in small reinforced concrete bridges that were in their turn subjected to a diagnostic load test. In the obtained results, it was possible to verify the clear advantages associated with the application of the smart-FRP system related with installation cost, time reduction and performance enhancements. It should be noted that this system was also tested by the same authors as reported in [118] on a historical building where the effectiveness of Brillouin strain monitoring was verified even for reasonably weak strain levels.

In 2010, Villalba *et al.* implemented the OBR system on the bottom surface of the slab of a newly built highway viaduct in Barcelona for its monitoring during the bridge load test, thus marking the first time that a distributed sensing method with millimetric spatial resolution was used for load testing of a real, newly built concrete bridge [119,120]. 

In order to take advantage of the extended and global range of DOFS and at the same time achieve a high sensitivity, Zhao *et al.*, implemented a multiscale fiber optical sensing network with both FBG- and BOTDA-based sensors [121]. A more comprehensive information of the structure was reported thanks to the DOFS technique by easily obtaining the overall stress condition of the structure.

Regier and Hoult employed DOFS technology in the monitoring system of a reinforced concrete bridge (the Black River Bridge) outside of the town Madoc, Ontario (Canada) [122]. The OBR-based DOFS obtained good results that were validated by other instrumentation such as strain gauges. Furthermore, the results obtained by the distributed fiber optics were then used to calculate deflections that compared well with measurements obtained by displacement transducers sensors. This confirmed that, provided the boundary conditions, measurements of distributed fiber optic sensors can be used to obtain structural deflections. Despite effectiveness of the employed DOFS in locating cracks and the strains related with crack widening during loading, the strain measurement in the locations near the cracks was reported as a challenge due to the fiber robustness properties. Additionally, the subject of temperature compensation was deviated through the acquisition of data over short term periods, however further research was advised for this topic regarding long-term monitoring and also for techniques that would make possible the accurate conversion of strain measurements at a crack to a crack width.

**Figure 26:**
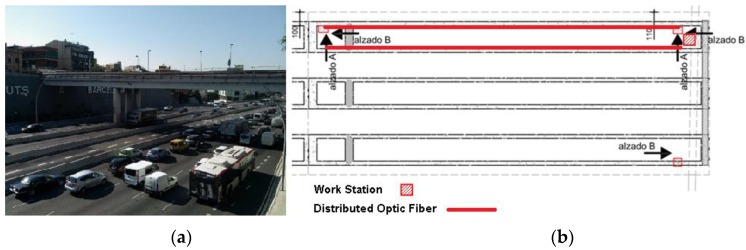
Sarajevo Bridge at Barcelona (**a**) and general scheme of DOFS monitoring scheme (**b**) (adapted from [123]).

More recently, Rodríguez *et al.*, instrumented one span of a bridge in Barcelona, Spain with an OBR system, glued without protective coating to the inner surface of the slab girder of the bridge, in order to monitor this structure during a deck enlargement rehabilitation, Figure 26. This process was conducted through several months, spanning from summer to winter, so consequently the topic of temperature influence in the measurements was a relevant issue that needed to be addressed [123].

With this implemented system it was possible to control the strain variation on the deck due to different and various stages of the widening process, ensuring in this way its structural safety.

### 3.5. Dams

Like bridges, also dams constitute an ideal area of application of DOFS monitoring due to their massive dimensions. Thévenaz *et al.* was some of the first to assess the implementation of Brillouin distributed fiber optic sensors for the monitoring of a dam [112]. An increase of the power capability of the hydroelectric plant associated with the Luzzone dam in the Swiss Alps was achieved by stacking new 15 m × 10 m concrete slabs for a 3 m thickness. In this way, a DOFS was installed during the pouring of the concrete in a dense mat geometry in order two provide a 2D temperature distribution of the slab. It was able to observe that after several weeks despite that the outer regions had a relatively stable low temperature, it took a lot of time to cool the central area that reached temperatures of up to 50 °C and that otherwise would have gone unnoticed. These measurements proved to be reliable even for the demanding environment present, including snow, dust and great temperature deviations.

**Figure 27:**
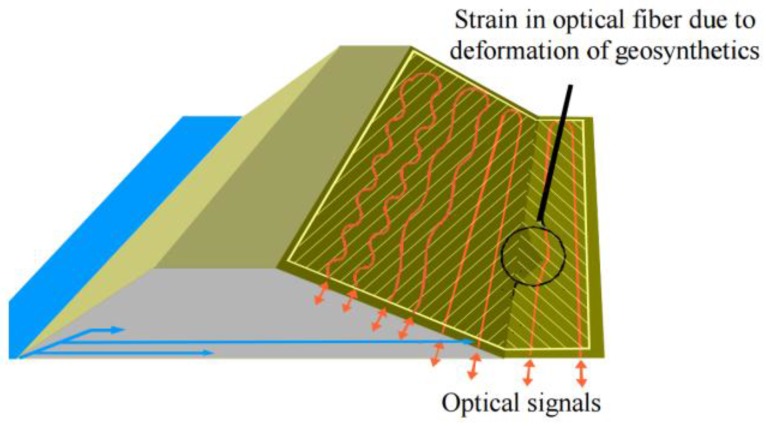
River dike with sensor-based geosynthetics [26].

Furthermore, the German Federal Ministry of Education and Research started a national research program named “Risk Management of Extreme Flood Events” (RIMAX) due to extreme floods in two large German rivers (the Oder in 1997 and the Elbe in 2002) in order to come up with smart monitoring systems that would be able to detect incipient effects of failure of hydraulic engineering structures. The main idea was to integrate FOS into geosynthetics that would be applied in the stabilization of geotechnical structures such as dams or dikes as seen in Figure 27.

Another case of dam monitoring using a commercial system named DiTeSt based on BOTDA technology is reported by Inaudi and Glisic for the Plavinu dam in Latvia [124].

### 3.6. Examples in Other Fields

The most sought out applications of DOFS were reviewed above, nonetheless, this technology, due its remarkable versatility, has found a wide range of potential applications that have been studied and developed in the past years and that are presented below.

**Figure 28:**
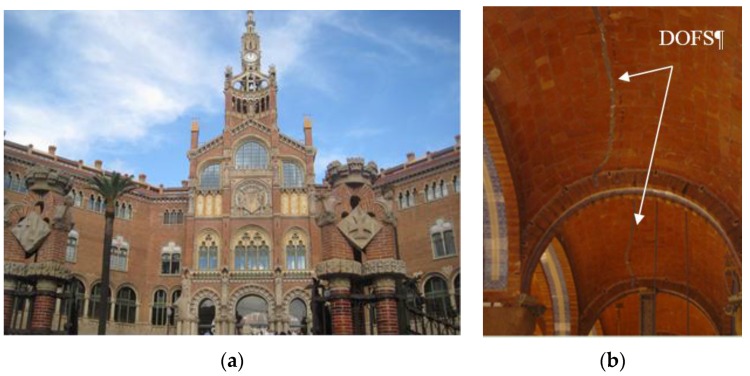
Sant Pau Hospital, Barcelona (**a**) and DOFS installed on the masonry vaults (**b**) (adapted from [123]).

For instance, historical buildings play an important role in the cultural heritage of any society; therefore the collection of data about their structural behavior is paramount in order to maintain such structures. The field of discrete fiber optic sensors has found a wide range of applications in this area. The same has not been observed with DOFS, except for the already briefly mentioned studies mentioned in [118] and [123]. In the latter, they implemented an OBR system during the replacement of two columns, for the monitoring of the adjacent upper slab of Sant Pau Hospital (a UNESCO World Heritage Site, Figure 28) in Barcelona [123]. This system allowed the successful assessment of the structural stability and safety of the slab by analyzing the slab stress redistribution in a distributed and continuous way (both in time and space).

**Figure 29:**
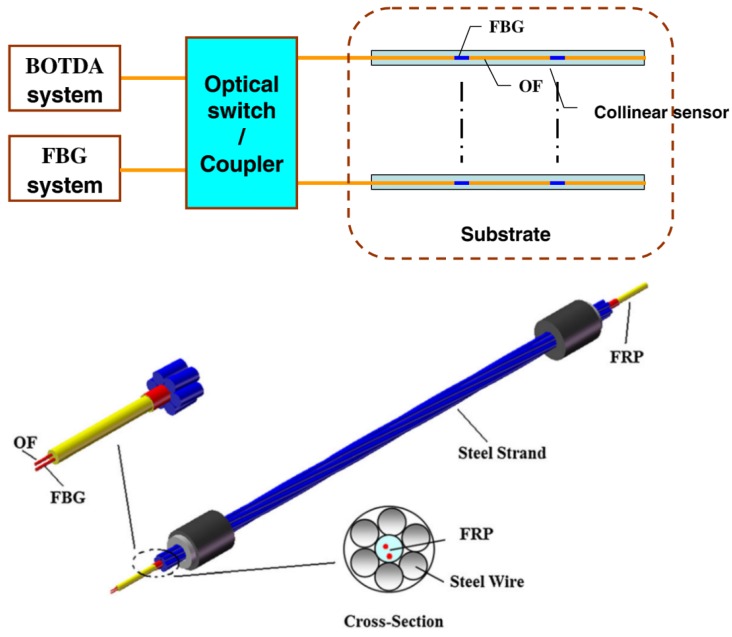
Proposed novel smart steel strand with FBG and BOTDA on a single fiber (adapted from [125]).

Monitoring of loss of prestress in concrete beams is of great importance. Lan *et al.* proposed a novel smart steel strand that combined BOTDA and fiber Bragg grating sensors on a single optical fiber by embedding it into a 5 mm diameter fiber-reinforced polymer (FRP) rebar [125] (Figure 29). 

For this, they tested several prestressed RC beams simultaneously monitored by BOTDA/R and FBG sensors that were then compared the results with measurements from more conventional sensors. Their results confirmed the viability of the proposed sensing system showing not only the spatial distribution of prestress loss, but also its time history for both construction and in service phase. 

Casas *et al.* also reported the monitoring of a cooling tower in Spain with an OBR sensor system [120]. Two main vertical cracks had appeared in this structure and a DOFS system was implemented, glued to the surface of the structure, in order to monitor the structural behavior before and after crack repair. This allowed the increase of the lifetime of the monitored structure by showing the origin of the main cracks and the means for implementation of the appropriate repair methods.

**Figure 30:**
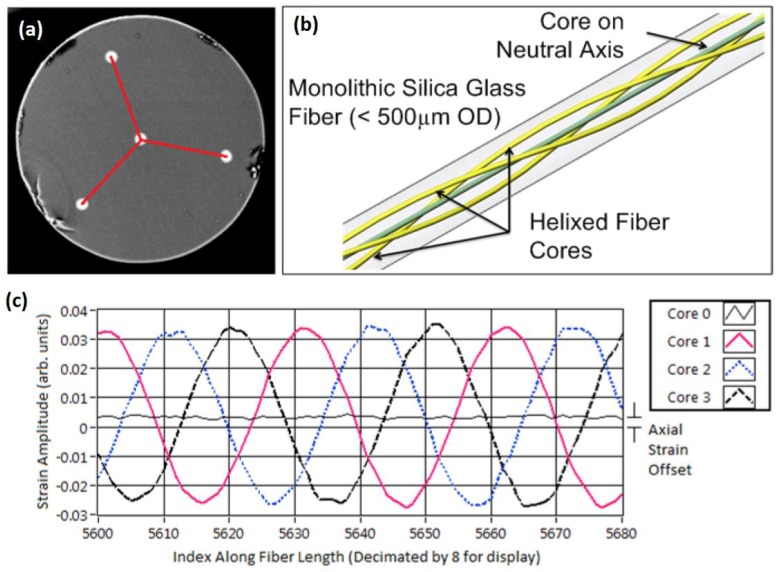
Illustration of shape sensing using helixed multi-core optical fiber: (**a**) SEM micrograph of shape sensing fiber, sensing triad in red; (**b**) Illustration of helical cores along length of fiber; (**c**) Typical four-core strain response to external curvature [126].

Furthermore, with the advance of new materials more and more flexible structures are being developed either for civil, mechanical and aerospace applications. These structures are designed for critical loads that are associated with dynamic loading conditions and therefore the monitoring of the distributed shape of flexible components provides important information during design, testing and operation. In this way Lally *et al.* proposed a novel, helically-wound geometry fiber (Figure 30), that uses Rayleigh scattering-based OFDR and is able to measure distributed curvature, twist, longitudinal strain/temperature and 3D shape along its entire length [126]. For more information about the behavior of this sensor the reading of the corresponding patent is advised [127].

**Figure 31:**
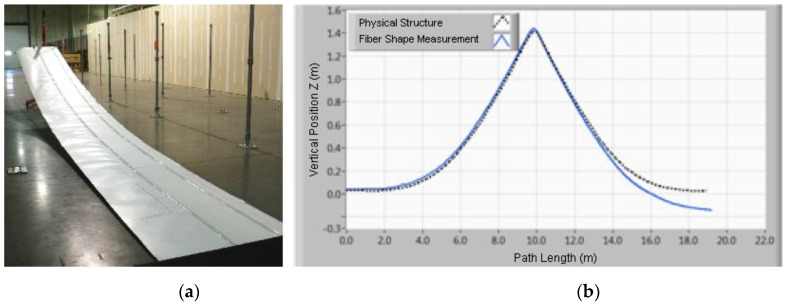
(**a**) experimental setup; (**b**) obtained results (adapted from [126]).

To test this new sensor, the authors conducted a laboratory test that consisted on the application of this fiber on a 10 m long flexible structure that would be subject to a 1.4 m translation at one of the ends of this structure as seen on Figure 31a.

The obtained results (as seen on Figure 31b were very promising, presenting a RMS error of 5.8 cm being that the biggest difference is verified at the end of the fiber due to a systematic error in the measurement of twist. Another tested flexible structure where a sensing fiber was implemented in a convoluted path in opposition to the U-shaped one seen in Figure 31 provided better results regarding this detail.

### 3.7. Dynamic Capabilities

The capability of dynamic strain response measurements is of great importance for the evaluation of structural fatigue that results from seismic activities and material deterioration [128]. Since the first and most practiced implementations of DOFS were traditionally focused on long range performance, the measurements were then limited mainly to static or quasi-static measurements. Dynamic measurements are more challenging to obtain since they require wide frequency range scanning and large scale averaging to improve weak signals [13]. However, some progress has been made in the past few years in making DOFS technology truly dynamic with sampling rates approaching tens of kHz [48], especially with Brillouin-based techniques [129,130,131,132,133,134].

**Figure 32:**
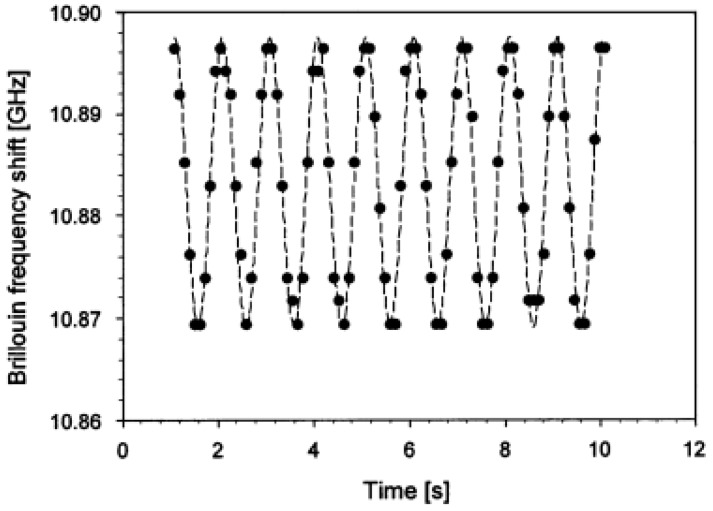
Brillouin frequency shift measured as a function of time, at a sampling rate of 8.8 Hz, when a 1 Hz vibration is applied [135].

The first study, reported by Hotate [135] presented a novel correlation-based continuous-wave technique for high spatial resolution and distributed dynamic strain measurements with stimulated Brillouin scattering sensing. A fully measured dynamic strain from a 5 cm vibrating section was reported with a strain accuracy of about ±38 µε at a sampling rate of 8.8 Hz as seen in Figure 32.

Later, Song and Hotate demonstrated 200 Hz distributed sensing (at 1 kHz sampling rate) over a 20 m single fiber measurement with a 10 cm spatial resolution [136] and strain distribution along a 100 m length fiber at 20 Hz sampling rate with 80 cm spatial resolution [137]. Most notably, Minardo *et al.* performed an experimental modal analysis of a cantilever beam with a BOTDA setup operated at a fixed pump probe frequency shift. This technique allowed the maximum acquisition rate of 108 Hz during the distributed strain measurements of the vibrating beam. The three first bending mode shapes were obtained with the deployed system and validated by a FEM model analysis [138]. Bao *et al.* also demonstrated the field application of impact wave detection on a concrete deck excited by the passing of a car at a frequency up to 300 Hz with a BOTDA-based sensor [139].

For Rayleigh scattering-based OTDR, the first reported vibration sensor was achieved through polarization OTDR achieving 10 m spatial resolution and 5 kHz event detection [140] and the best spatial resolution of 0.5 m over 1 km sensing length was achieved with coherent detection of phase OTDR at 8 kHz [141]. 

**Figure 33:**
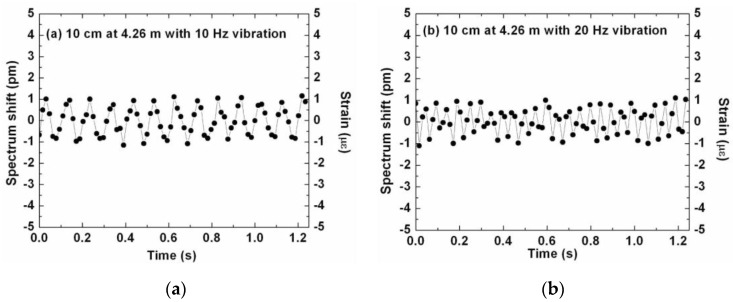
Time-domain Rayleigh backscatter spectrum shift (applied strain) with 10 cm spatial resolution when (**a**) 10 Hz and (**b**) 20 Hz sinusoidal voltage is applied to the PZT tube. [142].

Regarding Rayleigh-based OFDR, Zhou *et al.* performed some experiments in order to investigate the feasibility of distributed dynamic measurements with this technique [142]. The dynamic strain behavior was induced by wrapping a 20 cm fiber section on a lead zirconate titanate (PZT) tube with a diameter of 3 cm. The measurement of vibration frequency up to 32 Hz with a spatial resolution of 10 cm up to the total length of 17 m was demonstrated [142] as presented in Figure 33.

These examples showcase the potential of DOFS application for dynamic measurements in civil engineering structures. Nonetheless, this research topic is still in a very early development stage and more field applications need to be performed.

## 4. Challenges When Using Distributed Optical Fibers

As happens with any type of monitoring sensing system there are different challenges associated with the application of DOFS systems like the aforementioned limited spatial resolution and monitored length range. However, probably the major practical concern with fiber sensors in SHM of civil engineering structures is to assure that the sensor itself is not damaged during the installation or measurement process. This is not easily achieved due to the great fragility of bare fibers. 

As seen in the different applications showcased in this article, a comparison of distinctive coatings and protections for the optical fiber when applied to various structures is of extreme importance [42,83,84,86]. By using a relative thick coating, the process of implementation of the sensor becomes easier and the probability of rupturing the fiber is diminutive, however, the complete stress and strain transfer from the monitored structure to the sensor is not guaranteed. On the other hand, in applications where the coating is very thin or even non-existent, such as the cases of [89,91,120,123], the quality of the measured strains is improved, but at the same time, when applying the sensor to the structure (either embedded or glued to the surface of the structure) a great deal of attention and effort is required in order to not damage the sensor, implying also a strong limitation in long-term applications on exposed structures. This is a compromise that is specific to each implementation and should be carefully considered when planning any DOFS-based monitoring system.

Another concern related with the application of DOFS is the possibility of introducing bending stresses in the fiber during the field installation process, if sufficient care is not taken, which can be very detrimental due to the particularly weak scattering signals.

Additionally, in concrete structures, for bonded applications, the roughness of the concrete surface and the heterogeneity due to the presence of aggregates of several sizes leads to an even greater challenge. Cleaning of the grease and additional unwanted particles in the areas were DOFS are intended to be bonded, jointly with a pre-treatment to smooth the surface (brushing) is vital to achieve a good bond between the sensor and the instrumented structure. Also, when analyzing the measured data, low peaks and discontinuities appear due to those same surface irregularities and concrete flaking and spalling so special care should be taken in this step too in order to eliminate false assumptions.

Finally, when taking in account the use of DOFS systems in a damage/deterioration SHM system, it is important to imply a certain amount of redundancy into the sensor network in order to guarantee relevant results even after the failure of one of the sensors.

## 5. Conclusions

During the past decades, journals and project reports have shown that SHM practice has now become a well-established and mature subject. Furthermore, and within this topic, OFS has suffered an exponentially increase of interest and as a result, several studies and applications have been presented in the past years. However, examples of the distributed potential of this technology are still in a relatively early practice stage. As a result, in the present paper, the topic of distributed fiber optic sensing in SHM practice was widely discussed and reviewed, in particular, the applications in civil engineering structures.

First the concept of SHM and the role of fiber optics sensors were presented. Then the background theory associated with the most used distributed sensing techniques were described and elaborated, being these divided between time domain reflectometry and frequency domain reflectometry. Afterwards a general review of the state-of-the-art applications of different DOFS in geotechnical structures, pipelines, bridges and dams was shown. This was complemented by the state-of-the-art of laboratory works that led to field applications and the most recent researched developments of this technology in various areas. It is worth mentioning that the applications found on this paper only cover the sensor design and basic implementation techniques and performance. For further information regarding each of the applications, the reading of the respective references is advised. Finally, a brief summary of the most relevant challenges associated with the application of DOFS in SHM was presented.

In order to completely establish this technology as a viable solution for civil engineering SHM applications there’s still a lot to be studied and developed. The attempt to enhance the resolution of these sensors is something that should be continued in future works in order to create better damage monitoring solutions based on these sensors. Additionally, the identification of the most appropriate coatings and installation methods for each implementation should be further investigated in order to ultimately provide general guidelines for the use of this technology in civil engineering SHM. 

As seen in this article, DOFS-based systems can be deployed in different applications which showcase its impressive versatility, but also stress the importance of adapting this system to each solution. The choice of the most appropriate DOFS technique is an obvious result of some parameters such as the size of the structure, spatial resolution, acquisition speed and so forth. While Brillouin- based techniques can easily cover great lengths and therefore acquire global information about a structure, their resolution is not ideal for damage identification. Some attempts to improve this resolution have been made and are presented in this review. However, a more cost-effective technique must be developed in order to achieve high spatial resolution based on Rayleigh OFDR sensing. This is still a recent and developing technology that nevertheless is anticipated to have an important role in structural health monitoring in the near future, if correctly developed and harnessed.

## Figures and Tables

**Table 1:** Performance of distributed and quasi-distributed sensing techniques.

Sensing Technology	Transducer Type	Sensing Range	Spatial Resolution	Main Measurands
Raman OTDR	Distributed	1 km [73]	1 cm [73]	Temperature
		37 km [74]	17 m [74]	
BOTDR	Distributed	20–50 km	≈1 m	Temperature and Strain
BOTDA	Distributed	150–200 km [13]	2 cm (2 km) [75]	Temperature and Strain
			2 m (150 km) [76]	
Rayleigh OFDR/OBR	Distributed	50–70 m [11]	≈1 mm [66]	Temperature and Strain
FBG	Quasi-distributed	≈100 channels	2 mm (Bragg length) [21]	Temperature, Strain and Displacement
